# 

**Published:** 1877-08

**Authors:** 


CONTENTS.

| | PAGE |
|---|---|
| MORAL MEDICINE . . . . . . | 211 |
| LIFE AND DEATH . . . . . | 212 |
| THE INSTINCT OF APPETITE . | 212 |
| THE ACHIEVEMENTS OF SCIENCE | 215 |
| WE WRONG OUR DAUGHTERS | 217 |
| SCIENCE AIDING JUSTICE . . | 218 |
| THE BEST INHERITANCE . . | 221 |
| SCIENCE CHRISTIANITY'S HANDMAID . . . . . . . . . | 221 |
| THE NICE YOUNG MAN . . | 225 |
| BEAUTIFUL HAIR . . . . . | 226 |
| COLD FEET . . . . . . . | 227 |
| CRYING BABIES . . . . . . . | 227 |
| GAS-Y . . . . . . . . . . | 228 |
| LIVING BEYOND ONE'S MEANS | 229 |
| A PERSONAL APPEAL . . . | 232 |
| CONSTIPATION . . . . . . . | 233 |
| LEADEN WATER PIPES . . . | 233 |
| THE HEALTH FOOD CO. . . . | 234 |
| DELIGHTFUL TRAVELING . . | 234 |
| CHECKING PERSPIRATION . . | 235 |
| HINTS TO TRAVELERS . . . | 236 |
| SUMMER COMPLAINTS OF CHILDREN . . . . . . . . . | 237 |

